# Decoding NAD+ Metabolism in COVID-19: Implications for Immune Modulation and Therapy

**DOI:** 10.3390/vaccines13010001

**Published:** 2024-12-24

**Authors:** Shixu Song, Jialing Gan, Qiuyue Long, Zhancheng Gao, Yali Zheng

**Affiliations:** 1 Department of Respiratory, Critical Care and Sleep Medicine, Xiang’an Hospital of Xiamen University, School of Medicine, Xiamen University, Xiamen 361101, China; 2 Institute of Chest and Lung Diseases, Xiang’an Hospital of Xiamen University, Xiamen 361101, China; 3 Department of Respiratory and Critical Care Medicine, Peking University People’s Hospital, Beijing 100044, China

**Keywords:** SARS-CoV-2, COVID-19, NAD+ metabolism, immunometabolic therapies, enzyme modulation

## Abstract

The persistent threat of COVID-19, particularly with the emergence of new variants, underscores the urgency for innovative therapeutic strategies beyond conventional antiviral treatments. Current immunotherapies, including IL-6/IL-6R monoclonal antibodies and JAK inhibitors, exhibit suboptimal efficacy, necessitating alternative approaches. Our review delves into the significance of NAD+ metabolism in COVID-19 pathology, marked by decreased NAD+ levels and upregulated NAD+-consuming enzymes such as CD38 and poly (ADP-ribose) polymerases (PARPs). Recognizing NAD+’s pivotal role in energy metabolism and immune modulation, we propose modulating NAD+ homeostasis could bolster the host’s defensive capabilities against the virus. The article reviews the scientific rationale behind targeting NAD+ pathways for therapeutic benefit, utilizing strategies such as NAD+ precursor supplementation and enzyme inhibition to modulate immune function. While preliminary data are encouraging, the challenge lies in optimizing these interventions for clinical use. Future research should aim to unravel the intricate roles of key metabolites and enzymes in NAD+ metabolism and to elucidate their specific mechanisms of action. This will be essential for developing targeted NAD+ therapies, potentially transforming the management of COVID-19 and setting a precedent for addressing other infectious diseases.

## 1. Introduction

COVID-19 has persisted as a global pandemic for over three years. With the continuous emergence of new variants, the pandemic remains in a low-level, wave-like transmission pattern. Although antiviral therapies continue to be the primary treatment strategy, immunotherapy has demonstrated significant efficacy, particularly in severe cases of COVID-19, with interventions such as JAK inhibitors and IL-6/IL-6R monoclonal antibodies showing promise. However, the response rates for these therapies remain relatively low, at approximately 20–40% [1,2,3,4]. This highlights the need to explore novel, more effective treatment strategies further. In this context, the role of immunometabolism has gained increasing attention, particularly for its potential in modulating immune cell functions and regulating inflammatory responses [5,6,7].

Nutritional metabolites, including amino acids, fatty acids, and carbohydrates, serve not only as essential nutrients but also as critical modulators of both innate and adaptive immunity. These metabolites intricately regulate immune responses by influencing energy metabolism, biosynthetic pathways, immune signaling, and cytokine production. For instance, glutamine is a vital energy source for immune cells, significantly modulating their activation and function. Its metabolite, glutathione, is a crucial intracellular antioxidant, maintaining cell redox balance. Clinical studies have demonstrated that oral supplementation with L-glutamine, in addition to standard therapy, can significantly reduce hospitalization duration and ICU admission rates in COVID-19 patients [8]. Intravenous administration of N-acetylcysteine has been shown to prevent the depletion of glutathione levels, thereby improving organ function and clinical outcomes in severe COVID-19 patients [9]. Furthermore, arginine participates in immune regulation through multiple pathways, such as serving as a substrate for nitric oxide synthase to produce nitric oxide and influencing immune cell proliferation and differentiation via the mTOR signaling pathway [10]. Oral supplementation with L-arginine and vitamin C has been found to enhance physical performance, such as walking endurance and muscle strength, in adults suffering from long COVID-19 [11]. In addition, dietary creatine supplementation can alleviate symptoms associated with long-term COVID-19, such as loss of taste, body pain, headaches, and cognitive impairments [12]. Researchers are increasingly focusing on modulating the metabolic state of patients during or after infection to reduce inflammation, enhance antiviral immunity, and improve overall physical function.

Our previous research identified dysregulated nicotinamide adenine dinucleotide (NAD+) metabolism in COVID-19 patients, characterized by decreased NAD+ levels and elevated adenosine concentrations. There was a marked upregulation of NAD+-consuming enzymes, such as PARPs and CD38, in severe cases. NAD+ is a crucial intracellular coenzyme involved in various biological processes, including cellular energy metabolism, redox reactions, DNA repair, and signal transduction. The metabolism of NAD+ is essential for the development, activation, and regulation of immune responses in immune cells. Maintaining NAD+ metabolism is, therefore, critical for preserving immune cell function and modulating immune responses; alterations in its metabolic state may impact the host’s defense mechanisms and normal immune responses to pathogens.

This review explores the intricate interplay between NAD+ metabolism, infection responses, and the host immune system. Furthermore, it seeks to evaluate the scientific rationale and clinical potential of targeting NAD+ metabolic pathways as a novel therapeutic strategy for COVID-19.

## 2. NAD+ Metabolism

### 2.1. The NAD+ Metabolic Axis and Its Regulation

**Figure 1:**
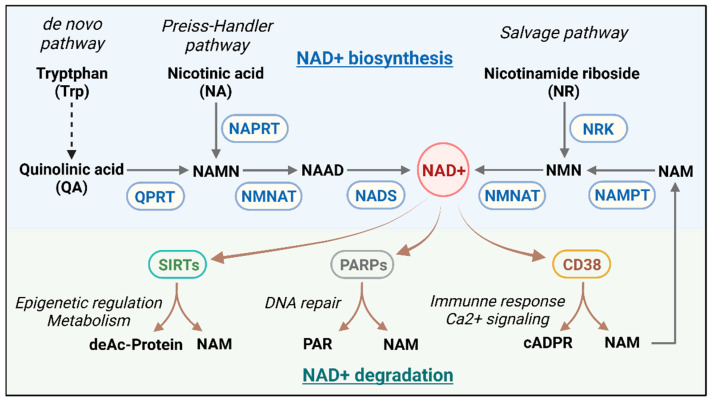
**Overview of NAD+ biosynthesis and degradation pathways.** The upper panel presents three pathways involved in NAD+ biosynthesis: the de novo pathway, the Preiss-Handler pathway, and the salvage pathway. In the de novo pathway, tryptophan (Trp) is converted into quinolinic acid (QA), which is processed into nicotinic acid mononucleotide (NAMN) by QPRT, eventually leading to NAD+ synthesis. The Preiss-Handler pathway converts nicotinic acid (NA) into NAMN through NAPRT, with subsequent conversion into NAD+ via NMNAT and NADS enzymes. The salvage pathway involves nicotinamide riboside (NR) being converted to nicotinamide mononucleotide (NMN) by NRK, which is then transformed into NAD+ by NMNAT. The lower panel illustrates the degradation and utilization of NAD+ by enzymes like sirtuins (SIRTs), poly (ADP-ribose) polymerases (PARPs), and CD38. These enzymes regulate diverse cellular processes such as epigenetic modifications, DNA repair, immune response, and calcium signaling. NAD+ is hydrolyzed by these enzymes, producing nicotinamide (NAM) as a by-product, which can be recycled back into the salvage pathway via NAMPT.

The NAD+ metabolic axis comprises a complex intracellular network involving NAD+ biosynthesis, consumption, and recycling (Figure 1). In mammalian cells, NAD+ synthesis primarily involves three key pathways: the de novo synthesis pathway, the Preiss-Handler pathway, and the salvage pathway. The de novo synthesis pathway starts with tryptophan metabolism, where tryptophan is converted through the kynurenine pathway into quinolinic acid (QA). This is then converted into nicotinic acid mononucleotide (NAMN) by quinolinate phosphoribosyltransferase (QPRT), eventually generating NAD+ through enzyme-mediated reactions. Alterations in this pathway’s activity are often associated with aging and age-related diseases [13]. The Preiss-Handler pathway uses nicotinic acid as a precursor, converting it into NAD+ via nicotinic acid phosphoribosyltransferase (NAPRT). This pathway mainly supports NAD+ biosynthesis by supplementing exogenous nicotinic acid intake. The salvage pathway is the primary mechanism for maintaining intracellular NAD+ levels. This route recycles NAD+ degradation products, particularly nicotinamide (NAM), converting it into nicotinamide mononucleotide (NMN) via nicotinamide phosphoribosyltransferase (NAMPT), followed by conversion into NAD+ through NMN adenylyltransferase (NMNAT). NAMPT is highly dynamic in mammals and plays a critical role in determining the rate of NAD+ synthesis [14]. It is closely linked to metabolic disorders such as obesity and diabetes [15,16]. Also, nicotinamide riboside (NR) can be converted to NMN by nicotinamide riboside kinases (NRKs), contributing to NAD+ biosynthesis.

The regulation of NAD+ metabolism involves various enzymes and signaling pathways, including sirtuins (SIRTs), PARPs, and CD38; each plays distinct roles in cellular processes like redox balance, DNA repair, cellular senescence, and immune responses. The major NAD+ consumption pathways include the following: SIRTs are NAD+-dependent deacetylases that consume NAD+ to deacetylate proteins, producing deacetylated proteins, NAM, and O-acetyl-ADP-ribose. This process regulates gene expression and cellular metabolism [17]. SIRTs are crucial in helping cells with metabolic stress and external stimuli, particularly by enhancing mitochondrial oxidative metabolism and stress resistance under various physiological and pathological conditions [18]. PARPs are activated in response to DNA damage, using NAD+ to synthesize poly (ADP-ribose) (PAR) chains that trigger DNA repair mechanisms. Excessive PARP activation can rapidly deplete intracellular NAD+ stores, impairing energy metabolism and affecting cell survival [19]. Moreover, CD38 is an NAD+-dependent cyclase that primarily converts NAD+ into cyclic ADP-ribose (cADPR) and NAM. As a calcium-signaling molecule, cADPR regulates intracellular calcium levels, impacting immune responses and metabolic regulation [20]. Overexpression of CD38 has been associated with aging and the onset of inflammation [21].

### 2.2. The Importance of NAD+ in Cellular Energy Metabolism and Redox Reactions

NAD+ is a critical cofactor in cellular energy metabolism and redox regulation by facilitating electron transfer. During glycolysis, where glucose is broken down into pyruvate, NAD+ is reduced to NADH. This NADH carries high-energy electrons that are subsequently utilized in mitochondrial metabolic processes [22]. In the oxidative decarboxylation reaction, pyruvate is converted into acetyl-CoA, reducing NAD+ to NADH. In the tricarboxylic acid (TCA) cycle, acetyl-CoA combines with oxaloacetate and is further oxidized to generate CO_2_, producing additional NADH and FADH_2_. These electron carriers feed into the mitochondrial electron transport chain (ETC) [23]. In the ETC, NADH transfers its high-energy electrons to Complex I, driving the translocation of protons across the inner mitochondrial membrane and establishing a proton gradient. This gradient powers ATP synthase to generate ATP while the terminal electron acceptor, oxygen, is reduced to water. Thus, NAD+ continuously cycles between oxidized and reduced forms, supporting efficient ATP production and cellular redox balance.

Beyond its role in energy metabolism, NAD+ is also crucial in mitigating oxidative stress. An imbalance between oxidant production and antioxidant defenses can lead to oxidative stress, such as when malfunctions in the electron transport chain result in excessive reactive oxygen species (ROS) generation [24]. NAD+ can be phosphorylated to form NADP^+^, a hydride acceptor, producing the reducing equivalent NADPH. This helps minimize electron leakage, thereby reducing the impact of oxidants [25]. Moreover, NAD+ regulates NAD+-dependent enzymes like SIRTs, which play pivotal roles in cellular responses to oxidative stress. SIRT1 deacetylates p65 to inhibit NF-κB transcriptional activity, lowering ROS levels, and deacetylates p53 to suppress apoptosis induced by oxidative stress [26,27]. SIRT1 can also upregulate Forkhead box O (FOXO) transcription factors and modulate peroxisome proliferator-activated receptor gamma coactivator 1-alpha (PGC-1α), enhancing mitochondrial antioxidant capacity and metabolic efficiency [28,29]. SIRT3, primarily localized in mitochondria, maintains the activity of antioxidant enzymes like superoxide dismutase 2 (SOD2) and glutathione peroxidase, thereby reducing ROS production and protecting cells under oxidative stress [30,31]. Thus, a deficiency in NAD+ levels can impair mitochondrial metabolism, increase oxidative stress, and reduce ATP synthesis, leading to inflammation and cellular damage [32,33].

### 2.3. Role of CD38 in Immunoregulation and Cell Signaling

We observed a significant increase in CD38 expression in COVID-19 patients, particularly those with severe disease. CD38, a multifunctional transmembrane glycoprotein, primarily catalyzes the conversion of NAD+ into cADPR and other nucleotide metabolites. It exhibits ADP-ribosyl cyclase and cADPR hydrolase activities, further hydrolyzing cADPR to produce ADP-ribose. These catalytic products function as critical intracellular second messengers that regulate cellular calcium (Ca^2+^) levels, thereby influencing essential immune functions like cell proliferation and T-cell activation [34,35].

CD38 is widely expressed across various immune cells, particularly in lymphocytes, dendritic cells (DCs), and natural killer (NK) cells [36]. Its expression is upregulated in response to inflammatory mediators and is often regarded as a marker of cell activation and differentiation [37,38]. In the immune system, CD38 serves dual functions: it is involved in the activation and differentiation of immune cells while also playing a crucial role in inflammatory responses and anti-infective defense. CD38 modulates cytokine release, cell adhesion, and the migration of immune cells to sites of inflammation [39,40]. During respiratory syncytial virus (RSV) infections, CD38 regulates Ca^2+^ mobilization and ROS production through its catalytic activity, impacting type I and III interferon responses, which are essential for antiviral and pro-inflammatory reactions [41]. Inhibition of CD38 enzymatic activity has been shown to reduce the expression of IFN-β, IFN-λ1, and ISG15 in infected DCs, thereby impairing the antiviral response.

Aberrant expression of CD38 has been associated with the progression of various cancers, including melanoma [42], glioblastoma [43], and hematologic malignancies [44]. In these tumors, CD38 not only regulates cancer cell survival and proliferation through metabolic pathways but is also linked to immune evasion mechanisms [45,46]. For instance, interactions between CD38 and its ligand CD31 facilitate cell adhesion, supporting the proliferation and migration of malignant hematologic cells [47,48]. By degrading NAD+ to produce adenosine, CD38 induces exhaustion in effector T cells via adenosine receptors [49].

In summary, the elevated expression of CD38 observed in severe COVID-19 patients is likely linked to its roles in inflammation and immune regulation. By further elucidating the function of the CD38-NAD+ metabolic axis in immune metabolism and signaling, we can explore novel therapeutic strategies to modulate immune responses, enhance anti-infective capabilities, and potentially offer new avenues for treating COVID-19 and other diseases.

## 3. NAD+ Metabolism in Infectious and Non-Infectious Diseases

### 3.1. NAD+ Metabolism in Infectious Diseases

The PARP family in humans comprises 17 members, with PARP1 being the most prevalent and extensively researched. PARP1 has crucial functions in programmed cell death, DNA repair, replication, and transcription. DNA strand breaks lead to elevated PARP1 activity, which results in NAD+ depletion and initiates an inflammatory response [50,51,52]. In hepatitis B virus-infected cells, heightened oxidative stress induced double-strand DNA (dsDNA) breaks, subsequently activating PARP1. Upon activation, PARP1 recognized the damaged dsDNA and promoted repair through the non-homologous end joining (NHEJ) pathway [53]. This process increased NAD+ consumption, suggesting a progressive NAD+ depletion. DNA ligation errors during repair occasionally caused virus–host DNA fusion events, potentially contributing to hepatocellular carcinoma development. In HIV-infected cells stimulated by TNFα, PARP1 overexpression led to a swift reduction in intracellular NAD+ levels. Notably, suppression of PARP1 expression was shown to inhibit viral replication, although the precise mechanisms remained uncharacterized [54]. Another study demonstrated that PARP1 inactivation in human monocyte-derived macrophages (MDM) also suppressed HIV replication. This inhibition occurred via disruption of long terminal repeat sequences (LTRs) and GTPase activity, which consequently reduced actin cytoskeletal rearrangements [55]. These findings underscore the multifaceted role of PARP1 in viral pathogenesis and its potential as a therapeutic target. Beyond PARP1, other PARP family members contribute to DNA repair processes during infections. For instance, disturbances in NAD+/NADH redox homeostasis in Mycobacterium tuberculosis (Mtb)-infected cells resulted in bacterial growth arrest and hindered mitochondrial aerobic respiration [56]. PARP9 expression was significantly upregulated in both Mtb-infected human and mouse cells. PARP9 modulated Mtb susceptibility by downregulating cGAS and mitochondrial oxidative stress-induced type I interferon (IFN) production, thereby reducing bacterial proliferation. Additionally, PARP9 interacted with DTX3L, a protein known to protect cells from DNA damage [57].

Similarly, the SIRT family comprises seven members, with SIRT1 emerging as a focal point in antiviral research. SIRT1 serves as both a metabolic sensor and a regulator of transcription factors, modulating pathways involved in glycolipid metabolism, DNA replication, and inflammatory responses [58]. In hepatitis C virus (HCV)-infected human hepatocellular carcinoma cells (HepG2), overexpression of the HCV core protein-induced oxidative stress, leading to a reduction in the NAD+/NADH ratio and downregulation of the SIRT1-AMPK pathway. This downregulation decreased the expression of genes associated with glucose and lipid metabolism, contributing to metabolic disturbances in hepatocytes [59]. In HBV infection, SIRT1 upregulated viral replication by modulating the transcription factor AP-1. Inhibition of SIRT1 with sirtinol significantly suppressed HBV DNA replication, indicating that SIRT1 inhibitors hold promise as a potential treatment for HBV [60]. Conversely, in Kaposi’s sarcoma-associated herpesvirus (KSHV) infection, a reduction in NAD+ levels lowered SIRT1 activity, which subsequently induced the expression of the transcriptional activator RTA, facilitating viral lysis and replication [61]. In septic mouse models, a marked decrease in NAD+ levels inhibited the NAD+/SIRT1 pathway in the hippocampus, while inflammatory pathways such as NF-κB and P38-MAPK were upregulated. Supplementation with NMN, a precursor of NAD+, restored NAD+/SIRT1 pathway activity, thereby attenuating the inflammatory response [62]. In addition to SIRT1, SIRT3 activity also relies heavily on NAD+ levels [63]. In macrophages infected with Mycobacterium tuberculosis (M.tb), the downregulation of SIRT3 leads to reduced expression of enzymes involved in central metabolism and components of the electron transport chain. This reduction results in increased mitochondrial ROS production and subsequent cell necrosis. Studies using Sirt3-deficient (Sirt3-/-) mice infected with M.tb further demonstrate that SIRT3 plays a protective role in defending the host against M.tb infection [64].

Finally, CD38, which is expressed in a variety of immune cells, is strongly induced to regulate NAD+ levels during infection and inflammation [65]. In HBV-specific CD8+ T cells, CD38 overexpression led to NAD+ depletion and the dysregulation of DNA repair mechanisms. As a result, the enzymatic activity of SIRTs was reduced, leading to mitochondrial dysfunction, increased ROS production, and DNA damage [66]. In HIV patients, increased CD38 activity in CD4+ T cells depleted NAD+, decreased SIRT activity, and impaired oxidative phosphorylation, ultimately causing mitochondrial dysfunction and T cell depletion. CD38 catalytic products also increased intracellular Ca^2+^, exacerbating mitochondrial oxidative stress and contributing to T cell depletion [67]. In RSV-infected mononuclear-derived dendritic cells (MDDCs), activation of the CD38-cADPR axis led to increased production of type I interferons, initiating an antiviral immune response. However, the precise role of the inflammatory response induced by Ca^2+^ channels activated by CD38 requires further investigation [41]. The changes in NAD+ levels in different infectious diseases are given in Table 1.

### 3.2. NAD+ Metabolism in COVID-19

NAD+ exhibits significant metabolic imbalances at various stages of COVID-19. During the acute phase, viral replication and cellular stress disrupt cellular energy metabolism, leading to an inflammatory response and elevated NAD+ consumption [68]. This reduction in NAD+ is identified as a risk factor for severe COVID-19 [69]. Additionally, the intense inflammatory response and oxidative stress during this phase exacerbate the depletion of NAD+, which in turn causes cellular metabolic disorders, impaired organ function, and respiratory failure, along with other complications [70]. Interestingly, chronic fatigue in long COVID-19 patients is linked to persistently low levels of NAD+ [71]. This deficiency affects cellular repair mechanisms, leading to chronic inflammation, mitochondrial dysfunction, and metabolic abnormalities [72]. The levels of NAD+ metabolites in the blood of COVID-19 patients decreased as the disease worsened. Concurrently, cytokines such as IL-6, IL-10, IL-8, M-CSF, and IL-1α showed elevated levels, suggesting that disturbances in NAD+ metabolism were closely associated with the host immune response [73]. On top of that, SARS-CoV-2 infection interfered with NAD+ metabolism in a mouse model, evidenced by increased expression of NAD+-depleting enzymes such as RARPs (RARP9, RARP10, RARP14) and the CD38 gene, along with downregulation of SIRT1 expression [74]. This suggested that, following SARS-CoV-2 infection, host-pathogen interactions may disrupt NAD+ metabolism by modulating the activities of these enzymes, leading to massive NAD+ depletion.

**Figure 2:**
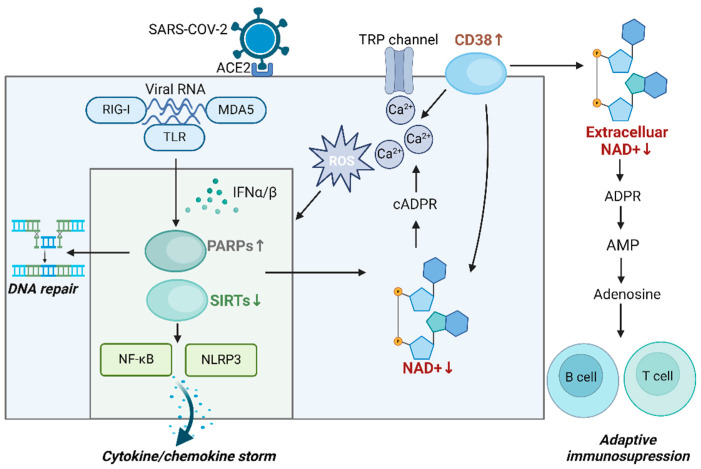
**Impact of changes in NAD+ metabolism on the immune response in COVID-19 infection.** Upon entry of SARS-CoV-2 into host cells via the ACE2 receptor, the viral infection induces DNA damage that activates PARPs, leading to increased NAD+ consumption and further cellular damage, thereby compromising immune cell resilience. This NAD+ depletion reduces SIRT activity, impairing its anti-inflammatory regulatory function and triggering a surge of cytokines and chemokines that disrupt the innate immune balance. Concurrently, upregulation of CD38 expression exacerbates NAD+ depletion, promoting inflammation through calcium signaling to enhance innate immunity while converting extracellular NAD+ into adenosine, suppressing adaptive immune responses.

Once SARS-CoV-2 binds to the ACE2 receptor, it enters the cell and releases its RNA genome [75]. Host cells recognize viral RNA through pattern recognition receptors, such as TLR, RIG-I, and MDA5, which activate the production of IFN, especially IFN-α and IFN-β [76] (Figure 2). This interferon signaling then triggers the transcription and activation of PARP1, which plays a role in DNA repair and inhibition of viral replication. However, overactivation of PARP1 reduces NAD+ and NMN levels, causing cellular metabolic dysfunction, cell death, and even tissue damage [68]. In addition, the excessive activation of PARP1 indirectly reduces SIRT1 activity [77]. This inhibition of SIRT1 disrupts energy metabolism homeostasis and activates NF-κB, which further induces inflammatory responses, contributing to the production of chemokine storms. Inhibition of SIRT1 also leads to NLRP3 overactivation, triggering a cytokine storm [58]. Inflammatory pathways regulated by SIRT1, such as NRF2/HMOX1, are also suppressed, which diminishes antioxidant defense functions and reduces the ability to inhibit viral replication [78]. Interestingly, SIRT5 was implicated in SARS-CoV-2 infection, as it interacted with NSP14, a viral protein of SARS-CoV-2, to promote viral replication [79]. In contrast, another study found that NSP14 interacted with SIRT5 to regulate host protein succinylation after SARS-CoV-2 infection, inhibiting viral replication [80]. Although these two studies present contradictory findings, they collectively suggest that SIRT5 regulates innate immunity during SARS-CoV-2 infection. The above highlights the complex relationship between NAD+ depletion, PARP activation, and the role of SIRTs in innate immunity during viral infections.

**Figure 3:**
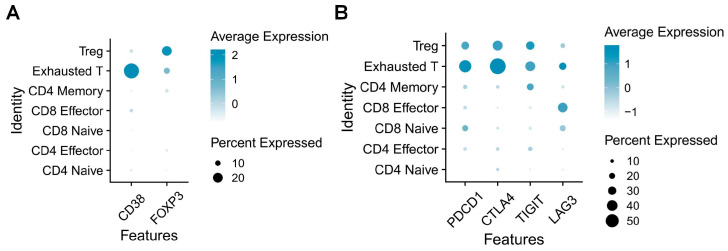
**CD38 and immune checkpoint expression patterns in T cell subsets of COVID-19 patients:** (**A**) Expression of CD38 and FOXP3 in different T cell subsets. (**B**) Expression of immune checkpoint molecules in different T cell subsets.

Along with the aforementioned immune modulators, CD38 may play a crucial role in innate and adaptive immunity during COVID-19. Studies have shown that CD38 expression on immune cells, such as T cells and monocytes, is elevated in COVID-19 patients, particularly in acute and critically ill individuals. This upregulation of CD38 expression may be associated with the severity of the disease [81,82]. Upon SARS-CoV-2 entries into host cells via the ACE2 receptor, angiotensin II (Ang II) binds to its receptor, activating CD38. This activation stimulates the release of Ca^2+^ from calcium channel proteins, leading to increased cytoplasmic Ca^2+^ concentrations, elevated ROS, and activation of type I IFN and IFN-stimulated gene (ISG) pathways. Subsequently, the NF-κB signaling pathway is activated, triggering NLRP3 activation and the release of large quantities of cytokines and chemokines, thereby inducing a cytokine storm [70]. CD38 is implicated in the innate immune response to SARS-CoV-2 infection by regulating Ca^2+^ homeostasis. Moreover, HLA-DR+CD38hiCD8+ T cells accumulated in severe cases of COVID-19 patients, exhibiting high levels of co-stimulatory and co-inhibitory molecules [83]. This suggested that these cells were in a state of simultaneous hyperactivation and depletion. As a marker of this population, CD38 may be a key regulator of T-cell depletion in COVID-19 patients. In our research on COVID-19 patients (data not published), we employed single-cell transcriptome analysis to delineate the profiles of immune cell subsets within Peripheral blood mononuclear cells (PBMCs). Our findings reveal a significant expansion of the exhausted T cell population in severe cases, characterized by elevated CD38 expression compared to other T cell subsets. This population also exhibits a regulatory T cell (Treg)-like phenotype and demonstrates increased expression of immune checkpoint molecules (Figure 3). Therefore, CD38 is also critical in modulating adaptive immunity during SARS-CoV-2 infection.

CD38 also regulates extracellular adenosine levels. It catalyzes the hydrolysis of NAD+ to cADPR, which acts as a second messenger to regulate Ca^2+^ homeostasis, indirectly influencing adenosine metabolism [84]. In addition, CD38 hydrolyzes extracellular NAD+ to ADPR, which generates AMP and adenosine [85]. Although the role of adenosine in regulating adaptive immunity in COVID-19 patients is not yet clear, its immunosuppressive effects have been demonstrated in conditions like sepsis. For example, adenosine inhibited macrophage-mediated bacterial killing by hydrolyzing ATP to adenosine [86]. Furthermore, in Sézary syndrome, a rare cutaneous T-cell lymphoma, T-cell activation induced adenosine production, which regulated T-cell immunosuppression and prevented excessive T-cell responses [87]. In diseases characterized by inflammation, such as COVID-19, extracellular adenosine may function as a negative immune checkpoint molecule [49,88]. Consequently, the elevated levels of CD38 in SARS-CoV-2 infection likely lead to increased adenosine levels, suppressing immune function. Modulating this pathway represents a potential therapeutic target for COVID-19.

### 3.3. NAD+ Metabolism in Non-Infectious Diseases

PARPs also play a critical role in NAD+-dependent DNA damage repair in non-infectious diseases. In a Drosophila model of Alzheimer’s disease (AD), reduced NAD+ levels were observed; however, introducing a RARP mutation increased NAD+ levels and improved mitochondrial function [89]. Likewise, another study demonstrated that treatment with NR, a NAD+ precursor, increased NAD+ levels in the brain of a mouse model of Alzheimer’s disease. This intervention led to a reduction in DNA release into the cytoplasm, which in turn reduced the aberrant activation of the DNA-sensing pathway. As a result, neuroinflammation levels decreased significantly, suggesting that PARPs could be targeted to enhance NAD+ levels as a potential therapeutic strategy for AD [90]. Moreover, in the context of BRCA-mutated ovarian cancer, a study found that the combination of DNA damage checkpoint kinase 1 (CHK1) inhibitors and poly (ADP-ribose) glycohydrolase (PARG) inhibitors acted synergistically to increase DNA damage. This, in turn, activated PARP1/2 to reduce NAD+ levels, causing metabolic stress and a decrease in tumor stemness [91]. The FDA-approved PARP inhibitors, such as olaparib, rucaparib, and niraparib, are applied to treat tumors with BRCA1/2-associated mutations, including ovarian and breast cancers with impaired homologous recombination (HR) repair. These inhibitors regulate DNA repair by recruiting MRE11 and NBS1, enzymes that play a crucial role in homologous recombination [92,93].

Apart from PARPs, SIRTs have been implicated in maintaining mitochondrial function and cellular energy balance, especially in non-infectious diseases. For example, in inflammatory bowel disease (IBD), depletion of NAD+ decreased SIRT1 activity and led to increased acetylation of PGC1α, contributing to mitochondrial dysfunction. However, treatment with NR restored the SIRT1-PGC1α axis, leading to improved mitochondrial function in mice with infectious colitis [94]. Similarly, in age-induced type 2 diabetes (T2D), mice exhibited significantly decreased NAD+ levels across several organs. Treatment with NMN normalized oxidative stress and inflammatory response pathways. Remarkably, SIRT1 deacetylated NF-κB, modulating hepatic insulin sensitivity and improving glucose intolerance and hyperlipidemia [16]. Furthermore, supplementation with NR increased NAD+ levels in mammalian cells and mouse tissues, thereby improving oxidative metabolism by activating SIRT1 and SIRT3. This helped prevent obesity induced by a high-fat diet [95]. In a mouse model of nonalcoholic fatty liver disease (NAFLD), SIRT2 regulated deacetylation and deubiquitylation of the fibronectin type III structural domain Fndc5, a process dependent on NAD+. This regulation reversed pathological processes such as steatosis, insulin resistance, mitochondrial dysfunction, and liver fibrosis in NAFLD [96].

In addition to its role in metabolic disorders, CD38 is also implicated in autoimmune diseases and tumors, primarily through its regulation of signaling pathways and mitochondrial oxidative stress. For instance, in Systemic Lupus Erythematosus (SLE) patients, CD8+ CD38hi T-cell subsets were increased, and NAD+ levels were depleted. CD38 activation led to the acetylation of EZH2, which inhibited SIRT1 activity, ultimately reducing the cytotoxic response [97]. Similarly, in chronic lymphocytic leukemia (CLL), CD38, a marker of poor prognosis, increased intracellular Ca^2+^ concentrations by converting NAD+ to ADPR and cADPR. This activation of chemokine receptors and integrins promoted proliferation and increased the invasiveness of CLL cells. Inhibition of CD38 activity blocked the homing of CLL cells from the bloodstream to lymphoid organs, suggesting that targeting CD38 could be a potential therapeutic strategy to inhibit CLL proliferation [98]. CD38 also played a crucial role in promoting oxidative stress in multiple myeloma (MM). Overexpression of CD38 in MM led to a generalized depletion of NAD+, triggering mitochondrial metabolic reprogramming and an increase in superoxide anion production, which contributed to increased oxidative stress. Interestingly, CD38 upregulation also enhanced the efficacy of NAD+-depleting agents in treating MM, though the exact mechanism of CD38’s involvement in MM remains unclear [99]. Additionally, in human non-small cell lung cancer (NSCLC) cell line A549 and HepG2, CD38 overexpression led to significant NAD+ depletion. This depletion activated multiple signaling pathways, including integrin, PI3K/AKT, and ERK/MAPK, promoting epithelial–mesenchymal transition (EMT). Supplementation with NAD+ precursors, however, inhibited STAT3 activity, reversed EMT, and ultimately inhibited tumor cell metastasis [100]. The alterations of NAD+ metabolism in different non-infectious diseases are detailed in Table 2.

## 4. NAD+ Metabolism, Aging, and COVID-19

### 4.1. NAD+ and Aging

Changes in NAD+ metabolism are closely associated with the aging process, with decreased NAD+ levels being a hallmark of various age-related pathological conditions [101,102,103]. Mitochondrial dysfunction is one of the hallmarks of aging [104], and studies have shown that defects in autophagy can deplete NAD+ [105]. Mechanistically, dysfunction in mitophagy, excessive mitochondrial ROS production, DNA damage, and overactivation of NAD+-consuming enzymes like SIRTs and PARPs lead to uncontrolled NAD+ consumption, eventually resulting in cell death. A decline in NAD+ levels compromises mitochondrial function, disrupting energy metabolism and contributing to chronic age-related diseases such as diabetes, obesity, and cardiovascular disorders [63].

NAD+ is a critical substrate for key enzymes, including SIRTs, PARPs, and CD38. SIRTs, in particular, play diverse roles in metabolic regulation and lifespan extension [106,107]. The SIRT2 gene, discovered in the 1990s, was one of the earliest longevity genes identified for its ability to extend yeast lifespan [108]. In 2020, He et al. linked SIRT2 to aging, chronic inflammation, and insulin resistance associated with overnutrition [109]. More recently, in 2023, Zhang et al. found that SIRT2 delays vascular aging and may serve as a potential therapeutic target for vascular regeneration [110].

When NAD+ levels decline, the activities of these enzymes are impaired, leading to cellular dysfunction and accelerated aging. Supplementation with NAD+ precursors such as NR and NMN has increased intracellular NAD+ levels, reversing certain aging characteristics and improving premature aging conditions in biological models. For example, Fang et al. demonstrated that NMN can protect neurons from pathological protein aggregation, improve memory in Alzheimer’s disease models, and extend the lifespan of aging-related disease models [111]. In addition, Mathieu et al. showed that supplementation with a novel NAD+ precursor, trigonelline, in *C. elegans* enhances mitochondrial activity, reduces age-related muscle atrophy, and extends lifespan. In mice, trigonelline supplementation improved muscle strength and reduced age-associated fatigue [112]. These findings highlight potential strategies for delaying aging and treating age-related diseases through regulating NAD+ metabolism.

### 4.2. COVID-19, Cellular Senescence, and Aging

The interplay between COVID-19 and aging has garnered significant attention, as increasing age is a major risk factor for severe outcomes and mortality in COVID-19 patients [113,114]. Epidemiological data show that COVID-19 mortality rates rise sharply with age [115,116]. In the United States in 2023, more than 70% of COVID-19 deaths were among individuals aged 75 and older, with the highest mortality rates observed in those over 85 [117]. Similarly, a study from Zambia analyzing clinical patients across four COVID-19 waves (2020–2022) found that the in-hospital mortality rate for those aged 60 and above was over three times that of younger patients [118]. The link between COVID-19 and aging is largely due to older individuals’ increased susceptibility and higher case-fatality rates. Biological aging involves a progressive decline in immune function, which reduces tolerance to pathogens and diminishes vaccine efficacy [119,120,121]. Aging is also associated with a chronic low-grade inflammatory state, predisposing the elderly to exaggerated inflammatory responses. This chronic inflammation promotes localized tissue damage and creates a pathological microenvironment that accelerates disease progression [122,123].

Interestingly, SARS-CoV-2 infection may accelerate the epigenetic aging clock and induce telomere attrition, thereby promoting epigenetic aging [124]. Additionally, the nucleocapsid (N) protein of SARS-CoV-2 inhibits DNA damage repair by binding to damage-induced long non-coding RNA, preventing the recruitment of the DNA repair protein 53BP1, which leads to cellular senescence and inflammation [125]. Tsuji et al. reported that SARS-CoV-2 infection induces a senescence-associated secretory phenotype (SASP) in infected cells, which can cause neighboring uninfected cells to enter a senescent-like cell cycle arrest [126]. SASP factors can trigger cytokine storms, destructive immune cell infiltration, endothelial inflammation, fibrosis, and microthrombosis [127]. Moreover, SASP factors can impair the immune system, contributing to immunosenescence characterized by increased cytokine levels such as IL-6, IL-RAT, TNF-α, and IL-1 [128]. This senescent phenotype persists even in patients without detectable active SARS-CoV-2 infection, resulting in chronic inflammation and various long-term symptoms, which contribute to the development of long-term COVID-19. Tove et al. found that senescence markers such as chitotriosidase and stathmin1 levels were elevated in the plasma of COVID-19 patients three months after hospitalization, correlating with pulmonary pathology [129]. The interplay between NAD+ metabolism, aging, and COVID-19, particularly in influencing the long-term course of the disease, remains to be fully elucidated.

## 5. Potential of Modulating NAD+ Metabolism in COVID-19 Treatment

### 5.1. Clinical Studies of NAD+ and Its Precursors as Therapeutic Interventions in COVID-19

In recent years, significant advances have been made in applying NAD+ in clinical research related to COVID-19. In the context of COVID-19 infection, reduced NAD+ levels are a pivotal factor contributing to virus-induced cellular stress responses, inflammation, and immune dysregulation. In 2020, Charles et al. analyzed lung cell samples from COVID-19 patients and found that viral infection induced the upregulation of PARP9, PARP12, and PARP14 expression, thereby accelerating NAD+ depletion, which led to various pathological issues [130]. This finding suggested that exogenous supplementation of NAD+ might enhance the immune system’s ability to clear the virus [131]. In preclinical studies, human aortic endothelial cells exposed to plasma from COVID-19 patients showed functional impairment, including reduced nitric oxide (NO) production and decreased NAD+ levels [132]. However, supplementation with NAD+ precursors such as NR and NMN significantly prevented this suppression and mitigated the increase in ROS triggered by exposure to COVID-19 patient plasma. Animal model studies have also demonstrated that NAD+ supplementation can ameliorate inflammation and cell death induced by SARS-CoV-2 infection. Mice infected with SARS-CoV-2 exhibited reduced pulmonary inflammatory infiltration and decreased cleaved caspase-3-positive cells after NAD+ treatment, substantially improving virus-induced gene expression and metabolic dysregulation [133].

Despite these promising results from preclinical studies, data on using NAD+ or its precursors in human clinical trials remain limited. Table 3 summarizes clinical trials in recent years that have explored the potential of targeting NAD+ metabolic pathways for COVID-19 therapy. In 2020, a clinical trial conducted in the United States reported that using a cocktail therapy consisting of NMN, zinc sulfate, betaine, and sodium chloride in high-risk COVID-19 patients led to rapid improvement in respiratory symptoms and inflammation markers [134]. Although this study lacked a control group, it laid the groundwork for further exploration of NAD+ metabolism modulation as a therapeutic approach for COVID-19. Subsequent large-scale clinical trials have demonstrated that supplementation with metabolic activators, including NAD+ precursors, significantly shortened the recovery time of COVID-19 patients, with notable improvements in laboratory markers reflecting heart, liver, and kidney function, such as alanine transaminase (ALT), creatinine, and lactate dehydrogenase (LDH) levels [135]. A clinical study focusing on patients with persistent acute kidney injury (AKI) secondary to COVID-19 revealed an increased urinary quinolinic acid-to-tryptophan ratio (Q/T ratio), which is indicative of impaired NAD+ biosynthesis [136]. Oral administration of NAM was associated with a reduced risk of renal replacement therapy or death, particularly in patients with KDIGO stage 2 or 3 AKI [137]. Additionally, computational studies predicting potential targets for SARS-CoV-2 structural and functional components have suggested that NAD+ precursors such as NMN and NR could interact with human ACE2 as well as viral proteins like spike (Spro), main protease (Mpro), and papain-like protease (PLpro). This highlights the potential role of NAD+ precursors as multifunctional therapeutic agents against COVID-19.

### 5.2. Targeting NAD+-Consuming Enzymes for COVID-19 Therapy

In addition to supplementing NAD+ precursors, modulating the activity of NAD+-consuming enzymes can also effectively increase NAD+ levels in the body. Excessive activation of PARPs in response to SARS-CoV-2 infection and the ensuing inflammatory response leads to disproportionate NAD+ consumption, further depleting its cellular levels. Inhibitors of PARPs, such as rucaparib and stenoparib, have been shown to suppress the replication of various SARS-CoV-2 variants and reduce the expression of pro-inflammatory cytokines [138,139]. Rucaparib exhibits a dual mechanism of action in COVID-19: it binds to the SARS-CoV-2 spike protein, inhibiting its interaction with the ACE2 receptor, thereby preventing viral entry into host cells, while also limiting NF-κB activation to induce an anti-inflammatory phenotype [139]. In vitro studies have also demonstrated that stenoparib exhibits a dose-dependent inhibitory effect on SARS-CoV-2 variants [140]. However, it remains uncertain whether inhibiting the PARP family could impair the ADP-ribosylation reactions in host cells, potentially compromising the body’s innate immune defenses.

It is well established that PARPs and SIRTs competitively regulate each other using their shared substrate, NAD+. SARS-CoV-2 infection suppresses the activity of certain sirtuins, such as SIRT1, whose activators are also being explored for COVID-19 treatment. Studies have demonstrated that resveratrol, a known SIRT1 activator, effectively inhibits SARS-CoV-2 replication in vitro [141]. Resveratrol enhances SIRT1-mediated anti-inflammatory pathways by suppressing TLR4/NF-κB/STAT signaling, which reduces the release of inflammatory cytokines from dysfunctional immune cells [142]. It also mitigates mechanisms linked to severe COVID-19, such as NLRP3 inflammasome activation, renin-angiotensin system dysfunction, and bradykinin–kallikrein system activation [143]. In a double-blind clinical trial involving outpatient COVID-19 patients, resveratrol treatment, compared to placebo, was associated with a lower hospitalization rate, reduced emergency visits related to COVID-19, and a decreased incidence of pneumonia [144]. Another SIRT1 activator, quercetin, has shown promise in enhancing SIRT1 expression and reducing NLRP3 inflammasome levels in COVID-19 patients, suggesting its potential as a therapeutic agent [145]. Other members of the sirtuin family may also exhibit unique anti-inflammatory and antiviral effects during SARS-CoV-2 infection; however, their specific roles and therapeutic potential remain poorly understood due to a lack of studies and approved clinical drugs targeting these enzymes in the context of COVID-19. CD38, another NAD+-consuming enzyme, has seen limited application in COVID-19 therapies. However, research has reported a connection between CD38+ Tregs and the immune response to mRNA COVID-19 vaccines, showing that the depletion of CD38+ Tregs could enhance the durability of immune responses to mRNA vaccines [146]. This finding may have potential clinical implications for immunocompromised patients, such as those with plasma cell disorders. Some studies have theoretically analyzed the use of CD38 inhibitors and monoclonal antibodies to improve COVID-19 outcomes by regulating NAD+ levels and immune function [82]. However, targeting CD38 in COVID-19 therapeutics may increase the risk of secondary infections. Research has shown that cancer patients treated with anti-CD38 monoclonal antibodies are more susceptible to bacterial infections and more vulnerable to viral infections [147]. Therefore, future research should assess the potential of targeting NAD+ consumption enzymes as immune modulators and be cautious of the risk of secondary infections that their inhibition may pose.

### 5.3. Restoration Strategies and Future Perspectives

NAD+ is a critical cofactor in cellular metabolism, particularly in energy production and redox balance, while also playing a vital role in regulating immune responses as a substrate for NAD+-consuming enzymes. During COVID-19 infection, studies have shown that NAD+ levels are often significantly depleted due to heightened inflammatory responses and increased activity of NAD+-consuming enzymes. Restoring NAD+ levels in COVID-19 patients may help alleviate inflammation. It could also reduce immune exhaustion and enhance cellular resilience, offering a novel therapeutic approach to strengthening host immune defenses and improving clinical outcomes.

Currently, the use of NAD+ precursors to restore NAD+ levels in COVID-19 patients has gained considerable attention. Recent studies suggest that these precursors may alleviate infection-related symptoms by modulating metabolic enzyme activity and reducing the production of pro-inflammatory cytokines. Additionally, targeting NAD+-consuming enzymes to mitigate NAD+ depletion is being investigated as a complementary therapeutic strategy. This approach may help restore the balance between immune activation and regulation, potentially modulating the activity of immune cells involved in severe COVID-19 responses.

Future research should explore targeted NAD+-based therapies, such as NAD+ precursor supplementation or enzyme modulators, combined with conventional antiviral or anti-inflammatory treatments. These combined strategies may provide a more comprehensive approach to managing COVID-19 symptoms and reducing complications. Additionally, employing in vivo imaging and molecular tracing technologies to monitor NAD+ metabolism in immune cells could pave the way for personalized NAD+ interventions, optimizing treatment timing and enhancing patient outcomes.

## 6. Conclusions

The review underscores the significance of NAD+ metabolism in COVID-19 therapy, linking its decline to increased cellular stress, inflammation, immune dysregulation, and aging. NAD+ precursor supplementation may bolster host defenses against SARS-CoV-2 and ameliorate the cytokine storm, with particular relevance for elderly patients. Future studies should focus on elucidating the roles of key NAD+-associated enzymes, optimizing NAD+ precursor therapies, and assessing their potential in managing COVID-19 and age-related conditions, paving the way for clinical adoption of NAD+-targeted strategies.

## Figures and Tables

**Table 1:** NAD+ metabolic imbalance in infectious diseases.

Enzymes	Disease	Model	Source	Observations	References
PARPs	HBV	HBV-infected HepaRG/HepG2	-	NAD+ consuming and PARP1 recognizes broken dsDNA and promotes DNA repair by (NHEJ) pathways	[53]
	HIV	HIV-infected U937 cells with TNFa. treatment	Flow Laboratories, Irvine, UK	PARP1 overexpression and NAD+ consuming	[54]
		HIV-infected MDM with PARP inhibitor treatment	University of Nebraska Medical Center, Department of Pharmacology and Experimental Neuroscience	suppression of HIV-1 replication by obstructing HIV-1 LTR activation	[55]
	MTB	Mtb H37Rv wt, NadE-DUC, and PpnK-DUC strains	-	NAD+ depletion and arrested growth of Mtb	[56]
		Parp9-/- mice	The Jackson Laboratory	increased susceptibility to Mtb infection mediated by type I IFN signaling	[57]
SIRTs	HCV	HCV core gene transfected HepG2 cells	-	decreasing NAD/NADH ratio and the activity of SIRT1, glucose, and lipid metabolism disorder	[59]
	HBV	HBV-transfected HepG2 cells	-	the upregulation of SIRT1 augmented HBV replication	[60]
	KSHV	KSHV-infected primary effusion lymphoma (PEL) cell line BCBL-1	-	Low NAD+ level disrupts viral latency by inhibiting SIRT1 function	[61]
	Sepsis	Septic mice induced by cecal ligation and puncture (CLP)	GemPharmatech Laboratory Animals (Nanjing, China)	Low NAD+ level and NAD+/SIRT1 pathway was inhibited	[62]
	MTB	J2 macrophages, BMDM, Sirt3-/- mice	University of Massachusetts Medical School and The Jackson Laboratory	SIRT3 downregulation, increased ROS, and cell death	[64]
CD38	HBV	CD8 T cells from patients with chronic active hepatitis	-	CD38 overexpression leads to NAD+ depletion and dysregulation of DNA repair mechanisms	[66]
	HIV	CD8 T cells from HIV patients	-	Increased CD38 activity promotes NAD+ consumption and exacerbates mitochondrial oxidative stress	[67]
	RSV	RSV infected MDDC	-	The increased production of type I IFNs activates CD38 and CD38/cADPR pathway	[41]

**Table 2:** NAD+ metabolic imbalance in non-infectious diseases.

Enzymes	Diseases	Models	Source	Observations	References
PARPs	AD	Aβ-Arc-expressing flies with PARP mutation	-	Increased NAD+ level and improved mitochondrial function	[89]
		APP/PS1 mice with NR treatment	The Jackson Laboratory	Aberrant activation of DNA sensing pathways and the level of neuroinflammation are reduced	[90]
	Ovarian cancer	OC cell lines OVCAR8 and SKOV3 exposed to CHK1 inhibition and PARG inhibitor	ATCC	Increased DNA damage, activation of PARP1/2, and decrease in NAD+ level	[91]
SIRTs	IBD	Mice subjected to experimental colitis	The Jackson Laboratory	NAD+ depletion, decrease in SIRT1 activity, and mitochondrial dysfunction	[94]
	T2D	High-fat diet-induced T2D mice and age-induced diabetic mice	The Jackson Laboratory	Decrease in NAD+ level, suppression of SIRT1 activity, and metabolic complications	[16]
	Obesity	High-fat diet-induced mice	Charles River	Decrease in NAD+ level, suppression of SIRT1/3 activity, and oxidative metabolism	[95]
	NAFLD	high-fat diet and methionine/choline-deficient diet-induced NAFLD mice with NR treatment	Sino-British SIPPR/BK Lab Animal Ltd.	Increase in NAD+ level and SIRT2 activity, reversion of hepatic steatosis, and steatohepatitis	[96]
CD38	SLE	CD8CD38high T cells from patients with SLE	ATCC	Decrease in MAD+ level and cytotoxicity	[97]
	CLL	CLL cells from Peripheral blood samples of patients with CLL	-	NAD+ depletion, increase in Ca^2+^ concentrations, and CLL aggressiveness	[98]
	MM	MM cell lines HMCL with CD38 overexpressing	ATCC or DSMZ	NAD+ depletion, NAD+ depletion, and mitochondrial metabolism reprogramming	[99]
	NSCLC/Liver Cancer	CD38+ A549/CD38+ HepG2	cell bank of the Chinese Academy of Sciences	Decrease in NAD+ level, promoting EMT	[100]

**Table 3:** Clinical studies of targeting NAD+ metabolism in COVID-19.

Trial ID	Interventions	Clinical Phase	Study Type	Results	References
NCT04573153	NR + serine + L-carnitine tartrate + N-acetylcysteine + hydroxychloroquine (CMA)	II/III	Randomized, placebo-controlled	In patients using CMAs, the time to complete recovery is significantly shorter, and plasma levels of proteins and metabolites associated with inflammation and antioxidant metabolism are significantly improved.	[135]
NCT04407390	NR 1 g/d	II	Randomized, double-blind, placebo-controlled	No results available yet.	
NCT04818216	NR 1 g/d	II	Randomized, double-blind, placebo-controlled	Patients in the nicotinamide riboside group had higher levels of NAD+ in whole blood.	
NCT05175768	NMN, NMN+L-leucine	Not applicable	Randomized, double-blind, placebo-controlled	No results available yet.	
NCT05038488	MIB-626	II	Randomized, double-blind, placebo-controlled	No results available yet.	
NCT04751604	Nicotinamide	Not applicable	Randomized, double-blind, placebo-controlled	No results available yet.	
NCT04910230	Nicotinamide	Not applicable	Randomized, double-blind, placebo-controlled	No clinical difference observed between therapy and placebo group.	
